# Unusual extraconal orbital location of a cavernous hemangioma

**DOI:** 10.3205/oc000204

**Published:** 2022-06-10

**Authors:** Gabriela Pacheco Callirgos, Francisco Javier Valentín Bravo, Pablo Panadero Meseguer, Víctor Manuel Asensio Sánchez

**Affiliations:** 1 Hospital Clínico Universitario, Ophthalmology Department, Valladolid, Spain; 2 Hospital Clínico Universitario, Pathology Department, Valladolid, Spain; 3 Hospital Clínico Universitario, Dacriology and Orbit Unit, Ophthalmology Department, Valladolid, Spain

## Abstract

Cavernous hemangioma is the most common benign orbital and vascular tumor in adults. It is mostly located intraconally. Nevertheless, when the location is extraconal, the displacement of the globe is opposite the tumor’s position. We describe an unusual presentation of this tumor in a 75-year-old female. The only symptom was the presence of epiphora. In the clinical examination, a mass was palpated on the lower orbital rim of the right eye. The magnetic resonance imaging (MRI) showed a well-circumscribed ovoid mass with a strong T2 hyperintensity and progressive contrast filling in T1. Excisional biopsy was performed, which confirmed the diagnosis of cavernous hemangioma. At five months of follow-up, there was no evidence of new symptoms.

## Introduction

Orbital cavernous hemangioma (OCH) is a vascular malformation and one of the most common primary orbital lesions in adults [1]. The incidence of OCH is 6% to 9% among orbital neoplasms [2]. It is a venous malformation characterized by slow growth and benign non-infiltrative behavior. Most OCHs are located in the intraconal compartment and in the orbit’s lateral part. A highly vascularized area favors their nutrition and development [3]. There is an unexplained predisposition to localization on the left side [4], and it occurs more frequently in women (60–70%), possibly due to a female sex hormonal influence [5].

Usually, the main symptom is a painless and unilateral progressive axial exophthalmos [6]. Furthermore, in less frequency, the patient could report diplopia, abnormalities of color vision and decreased vision. The clinical symptoms do not occur until the fourth or fifth decades of life in most cases [4].

Magnetic resonance imaging (MRI) and computed tomography (CT) are the primary imaging modalities used to evaluate orbital tumors and vascular lesions, with MRI being the most recommended because it avoids radiation [7].

The management usually is conservative, the observation is recommended in small hemangiomas, and surgery is considered in severe cases [8].

## Case description

A 75-year-old Caucasian female, with no personal history of hemangioma or other diseases, presented with a six-month history of the right eye’s epiphora. The ophthalmological examination showed a non-painful mass in the lower orbital rim on deep palpation. The lacrimal syringing was patent in both eyes.

**Figure 1:**
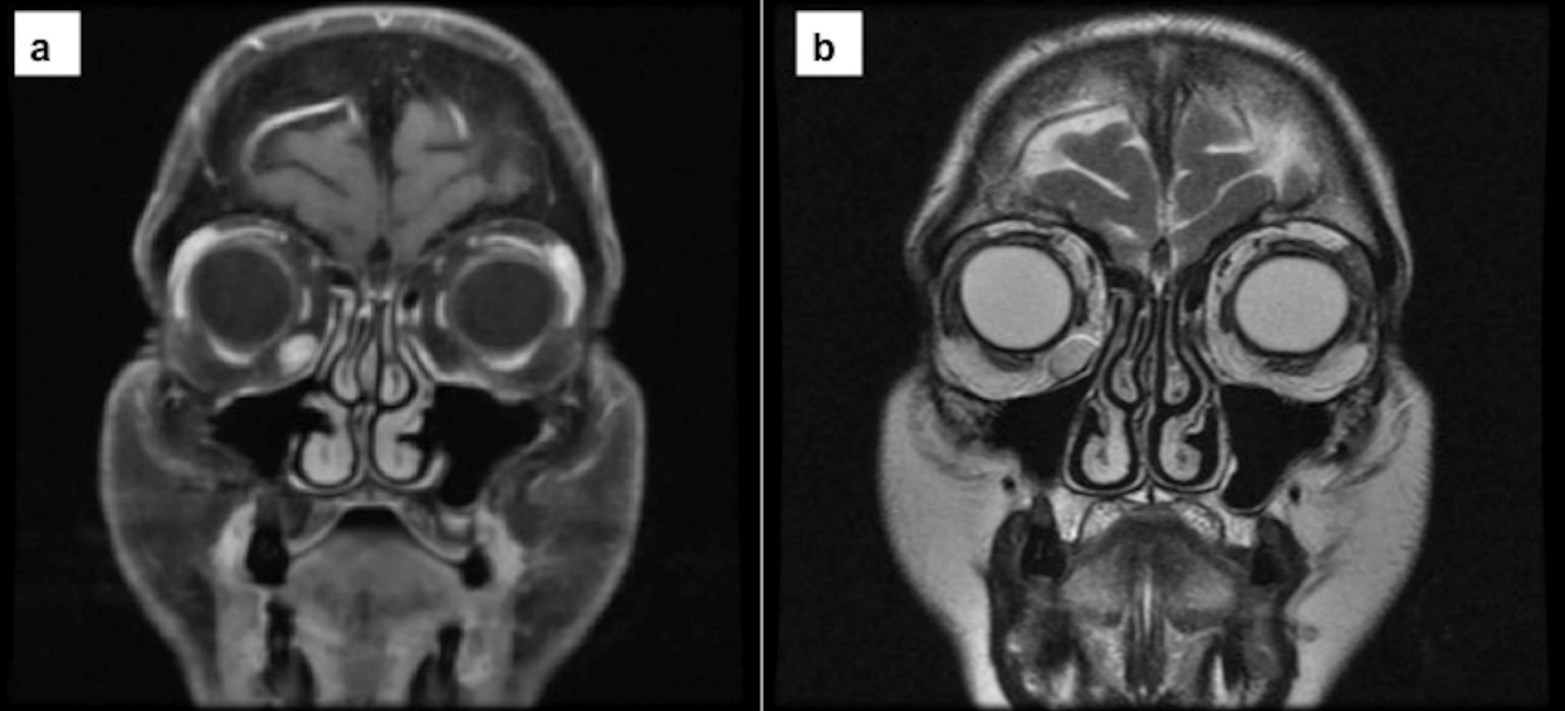
Magnetic resonance imaging of the coronal section. a) MRI – T1 gadolinium-enhanced showed a well-defined lesion in the inferior orbital rim that was enhanced with contrast. b) MRI – T2 showed a hyperintense lesion. The capsule is shown as well as low-intensity septation.

Magnetic resonance imaging (MRI) was performed. It showed a well-localized mass with circumscribed edges and progressive enhancement with gadolinium in sequence T1 (Figure 1a (Fig. 1)) and homogeneous enhancement in sequence T2 (Figure 1b (Fig. 1)).

A transcutaneous excisional biopsy was performed. An encapsulated blue, vascular, frambesiform tumor was identified in a deep plane. It was completely excised without intraoperative bleeding.

**Figure 2:**
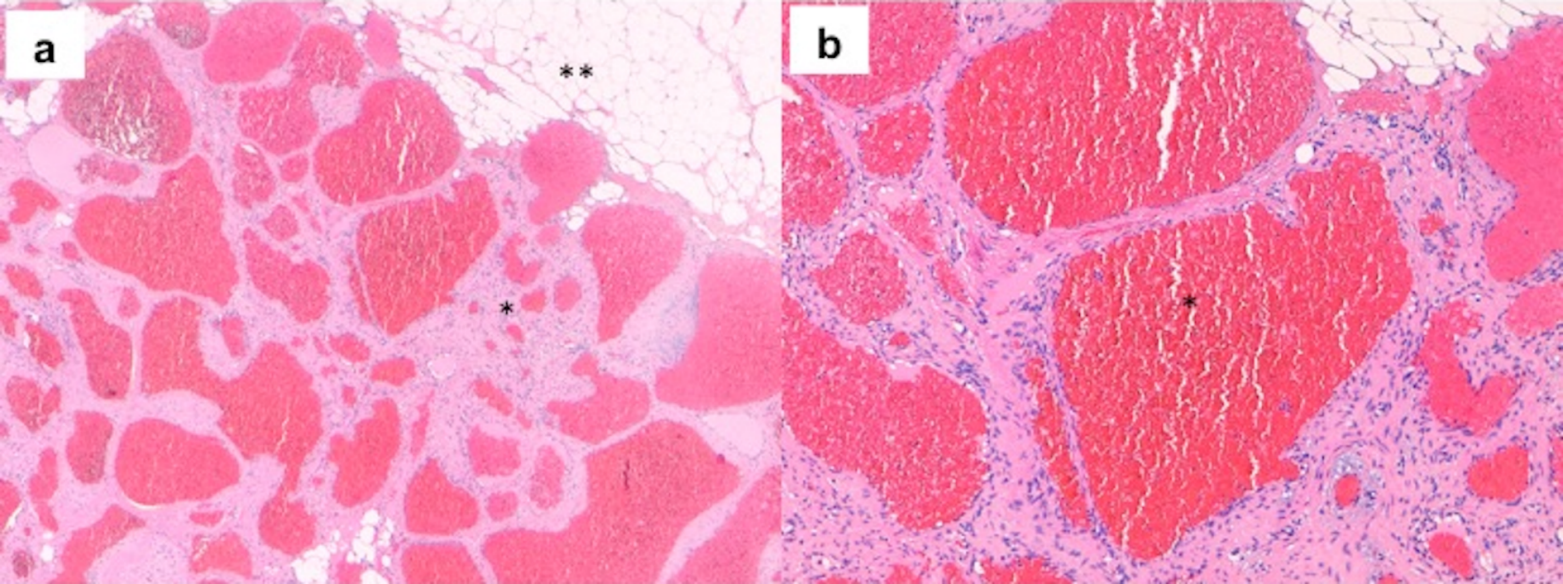
a) Fibrous benign stroma (*) with dilated cavernous vessels and mature adipocyte cells in the periphery (**), H&E x4. b) The vessels filled with red blood cells (*). The endothelium and the stroma showed no signs of malignancy, H&E x10.

The pathological anatomy findings confirmed the diagnosis. Grossly, it was a 1,2x1x0.3 cm reddish mass with an elliptical shape. On section, a hemorrhagic-like surface was observed. The microscopic report described dilated vessels with a cavernous appearance, and the lumen was occupied by red blood cells. The stroma was fibrous with a benign appearance, and adipose cells were observed in the periphery (Figure 2 (Fig. 2)).

Five months after surgery, the patient was stable and asymptomatic.

## Discussion

We described a case of cavernous orbital hemangioma with unusual localization and clinical presentation. It is the most common vascular lesion of the orbit in adults. More than 80% are located within the intraconal compartment, most commonly on the lateral side [9], [10], [11]. They occur more frequently in women (60–70%) during the fourth to fifth decades of life [12].

Moreover, extraconal hemangiomas are rare and are more challenging to diagnose in atypical areas.

Affected areas such as the nose and paranasal sinuses have approximately 100 cases reported in the literature [13], [14]. There is no literature about extraconal orbital hemangiomas located on the inferior rim of the orbit.

The maxillary hemangiomas can manifest as: facial mass, facial anesthesia or paresthesia, sinusitis, loose teeth, or oroantral fistula [15]. However, intraconal hemangiomas have slow growth with a gradual onset of symptoms like mass effect or proptosis.

The diagnosis is supported by MRI images, as shown in this case. Usually it appears isointense on T1 sequence with an enhancement after contrast administration with a progressive accumulation, and hyperintense on T2 sequence [16].

The definitive diagnosis is secured by the biopsy. In this case, the tumor showed large congested vessels separated by thin fibrosis septae and flattened endothelial cells [17]. The vascular lumen is filled with blood cells and variable regions of intralesional thrombosis, reflecting their vascular stasis [18].

The differential diagnosis of well-circumscribed orbital lesions include schwannoma, fibrous histiocytoma, hemangiopericytoma, and specific metastatic lesions [17].

The management of orbital hemangioma is usually conservative, and the surgery is reserved for cases that cause proptosis or optic nerve compression [18]. However, in this case, the aim of excisional biopsy was to rule out other malignant pathology for the unusual localization.

## Conclusion

To conclude, extraconal OCH is a relatively rare entity. A complete physical examination is necessary and essential to detect lesions that are not visible in the first instance. 

## Notes

### Acknowledgments

We would like to thank the Pathology and Radiology unit for the images.

### Competing interests

The authors declare that they have no competing interests.
